# DNA methylation subgroups and the CpG island methylator phenotype in gastric cancer: a comprehensive profiling approach

**DOI:** 10.1186/1471-230X-14-55

**Published:** 2014-03-28

**Authors:** Marie Loh, Natalia Liem, Aparna Vaithilingam, Pei Li Lim, Nur Sabrina Sapari, Eiram Elahi, Zuan Yu Mok, Chee Leong Cheng, Benedict Yan, Brendan Pang, Manuel Salto-Tellez, Wei Peng Yong, Barry Iacopetta, Richie Soong

**Affiliations:** 1 Cancer Science Institute of Singapore, National University of Singapore, Singapore, Singapore; 2 School of Surgery, The University of Western Australia, Perth, Australia; 3 Department of Epidemiology and Biostatistics, Imperial College London, London, UK; 4 Institute of Health Sciences, University of Oulu, Oulu, Finland; 5 National University Cancer Institute of Singapore, National University Health System, Singapore, Singapore; 6 Department of Pathology, National University Health System, Singapore, Singapore; 7 Centre for Cancer Research and Cell Biology, Queen’s University Belfast, Belfast, UK

**Keywords:** Methylation, Gastric cancer, Microarray, CIMP, GoldenGate

## Abstract

**Background:**

Methylation-induced silencing of promoter CpG islands in tumor suppressor genes plays an important role in human carcinogenesis. In colorectal cancer, the CpG island methylator phenotype (CIMP) is defined as widespread and elevated levels of DNA methylation and CIMP+ tumors have distinctive clinicopathological and molecular features. In contrast, the existence of a comparable CIMP subtype in gastric cancer (GC) has not been clearly established. To further investigate this issue, in the present study we performed comprehensive DNA methylation profiling of a well-characterised series of primary GC.

**Methods:**

The methylation status of 1,421 autosomal CpG sites located within 768 cancer-related genes was investigated using the Illumina GoldenGate Methylation Panel I assay on DNA extracted from 60 gastric tumors and matched tumor-adjacent gastric tissue pairs. Methylation data was analysed using a recursively partitioned mixture model and investigated for associations with clinicopathological and molecular features including age, *Helicobacter pylori* status, tumor site, patient survival, microsatellite instability and *BRAF* and *KRAS* mutations.

**Results:**

A total of 147 genes were differentially methylated between tumor and matched tumor-adjacent gastric tissue, with *HOXA5* and hedgehog signalling being the top-ranked gene and signalling pathway, respectively. Unsupervised clustering of methylation data revealed the existence of 6 subgroups under two main clusters, referred to as L (low methylation; 28% of cases) and H (high methylation; 72%). Female patients were over-represented in the H tumor group compared to L group (36% vs 6%; P = 0.024), however no other significant differences in clinicopathological or molecular features were apparent. CpG sites that were hypermethylated in group H were more frequently located in CpG islands and marked for polycomb occupancy.

**Conclusions:**

High-throughput methylation analysis implicates genes involved in embryonic development and hedgehog signaling in gastric tumorigenesis. GC is comprised of two major methylation subtypes, with the highly methylated group showing some features consistent with a CpG island methylator phenotype.

## Background

Gastric cancer (GC) is a complex disease that involves risk factors such as *Helicobacter pylori* (*H. pylori*) infection, family history of cancer, environment, diet and genetic susceptibility variants. GC typically has poor prognosis due to late clinical presentation at an advanced stage of disease [1]. Improvements in early detection via screening and the reduction of known risk factors such as chronic *H. pylori* infection and consumption of preserved/salted food [2-6] has resulted in significantly lower incidence rates in most parts of the world [7]. However, GC remains a major public health issue and is the fourth most common cancer type and the second leading cause of cancer death worldwide [8,9].

Transcriptional inactivation by cytosine methylation at promoter CpG islands of tumor suppressor genes is an important mechanism contributing to the development of human cancer. In several cancer types, subgroups defined by distinctive methylation patterns have been linked to features such as tumor size in breast cancer [10], tumor type in lung [11] and tumor histology in glioma [12]. The most well studied methylation-defined subgroup is the CpG Island Methylator Phenotype (CIMP) in colorectal cancer (CRC) first proposed in 1999 by Toyota et al. [13]. CIMP + CRC exhibit widespread CpG island methylation in gene promoter regions and are characterized by distinct clinical, pathological and molecular features. These include a higher incidence in females and in the proximal colon, poor histological differentiation and frequent association with microsatellite instability (MSI) and *BRAF* mutations [14,15]. A panel of five methylation markers has been proposed to standardize the evaluation of CIMP in CRC [16].

The existence of GC subgroups that are characterized by distinct methylation patterns and/or CIMP-like properties has been explored in several studies [17-26]. However, a standard panel of methylation markers has yet to be proposed for GC and technical issues remain concerning the use of non-quantitative analytical methods and the limited number of genes investigated for methylation. To consolidate knowledge on DNA methylation in GC, we recently performed a meta-analysis of 106 case–control studies that reported on the methylation of 122 candidate genes [27]. A total of 77 genes were found to be differentially methylated between tumor and normal tissue, including genes involved in apoptosis (*APAF2*, *BCL2*), cell cycle regulation (*p15*, *p16*) and DNA repair (*XRCC1*). Some studies alluded to the existence of CIMP by referring to a distinct subset of GC that exhibited a high frequency of concurrent gene promoter CpG island hypermethylation. However, the existence and phenotypic properties of CIMP in GC remain controversial, with major confounding factors likely to be the number and identity of CpG sites interrogated for methylation and the GC sample size and quality.

We previously demonstrated that the level of tumor cell content in GC has a major impact on the hierarchical clustering of methylation data [28]. We established that a tumor cell content of 70% was the minimum level required for the reliable analysis of methylation. In the current study, 60 GC samples underwent prior review by pathologists to ensure this minimum tumor cell content was met prior to methylation analysis using the GoldenGate Methylation BeadArray (Illumina) platform. This system allows simultaneous quantification of the methylation level at 1,421 autosomal CpG sites located within 768 cancer-related genes. The aim of our study was therefore to use a comprehensive genome-wide approach to investigate in an unbiased fashion whether methylation subgroups including CIMP occur in GC.

## Methods

### Tissue samples

Formalin-fixed and paraffin-embedded (FFPE) primary tumor and matched tumor-adjacent gastric tissue samples from 60 patients with GC were obtained from the Department of Pathology at the National University Hospital System, Singapore, under an institutionally approved protocol. The tumor samples were reviewed for their tumor content and scored in deciles independently by two experienced gastrointestinal pathologists (CLC, BY). All 60 cases included in the study had a tumor cell content of >70% [28]. This cohort has well-annotated clinicopathological information including age, gender, ethnicity, stage, location, tumor size, adenocarcinoma subtype, differentiation, Lauren classification, lymphoid invasion, perineural invasion, *H. pylori* status, history of chronic gastritis/atrophic gastritis/intestinal metaplasia/dysplasia, overall survival (OS), disease-specific survival (DSS), disease-free survival (DFS) and molecular features such as *BRAF* V600E, *KRAS* (codons 12 and 13) mutation and microsatellite instability (MSI).

DNA was extracted from 20 μm sections and verified for DNA quantity and quality as described earlier [29]. The sections were incubated for 3 days at 55°C in 200 μl of digestion buffer (10 mM Tris-hydrochloric acid, pH8.3; 1 mM EDTA; 0.5% Tween 20) and 45 μl of Proteinase K (20 mg/ml, Promega, Madison, WI) without prior dewaxing. The enzyme was inactivated by heating for 10 minutes at 94°C and then samples were centrifuged at 12,000 *g* for 10 minutes and stored at 4°C without further DNA purification. DNA quantity and quality were determined spectrophotometrically using the NanoDrop ND-1000 (Wilmington, DE). Five hundred nanograms of DNA was bisulfite-converted using the EZ DNA Methylation kit (Zymo Research, Orange, CA) as per the manufacturer’s instructions.

For the validation of candidates, frozen tumour and matched tumor-adjacent tissue from an independent sample series of gastric cancers were obtained from the National University Health System under an institutionally approved protocol. DNA was extracted using the DNeasy Blood and Tissue Kit (Qiagen, Hilden, Germany), quantified and bisulfite converted as described above.

### *BRAF* mutation, *KRAS* mutations and microsatellite instability (MSI)

Hotspot mutations in *BRAF* (V600E) and *KRAS* (codons 12 and 13) were detected using direct sequencing as described previously [30,31]. MSI was determined by analysis of 5 mononucleotide repeats, including BAT-25, BAT-26, NR21, NR22, NR24 and NR27, as reported by Buhard et al. [32], with tumors being defined as MSI when ≥3 markers showed instability.

### Illumina GoldenGate® methylation technology

Comprehensive DNA methylation profiling at 1,505 individual CpG loci contained within 807 genes using the Illumina GoldenGate Methylation Cancer Panel I (Illumina, San Diego, CA) was carried out as described by Bibikova and Fan [33]. Human sperm DNA and Universal methylated DNA (Chemicon, Temcula, CA) were included in each run as unmethylated and methylated controls, respectively. Hybridized arrays were scanned using the BeadArray Reader (Illumina). Normalization of background intensity was estimated from a set of built-in negative controls and subtracted from each methylation data point. To assess sample quality, only those samples having >75% loci with a detection p-value of less than 0.05 were included for analysis. The methylation level at each CpG site or the β-value was defined as the ratio of the methylated allele to the sum of the methylated and unmethylated alleles, and ranged from 0 (completely unmethylated) to 1 (completely methylated).

### Pyrosequencing

PCR was performed using 2 μl bisulfite DNA, 1 x reaction buffer with 1 mM MgCl_2_, 0.8 mM deoxynucleotide triphosphates, 1 unit of FastStart Taq DNA polymerase (Roche Diagnostics, Mannheim, Germany) and 400 mM each of PCR forward primer and a 1:9 mixture of PCR reverse primer and universal biotinylated PCR primer. PCR cycling consisted of incubation at 95°C for 4 min, 50 cycles of 95°C for 30 sec, 54°C for 30 sec and 72°C for 30 sec, followed by a final extension at 72°C for 1 min. Pyrosequencing was performed using the PyroMark annealing buffer (Qiagen) and PyroMark binding buffer (Qiagen), 3 μL Streptavidin Sepharose High Performance beads (GE Healthcare, Stockholm, Sweden) and 350 mM pyrosequencing primer on the PyroMark Q24 (Qiagen) according to manufacturer’s instructions. The PCR forward, reverse and sequencing primers, and pyrosequencing dispensation order were 5′-TTT GGA AGT TAG GAT TTT GG-3′, 5′-GGG ACA CCG CTG ATC GTT TAT CAA TAA AAA AAA AAC AAC CTC AA-3′, 5′- GTT TAT TTA GGG TTG TAA TGT TTT A-3′ and CTA CGA TCT GTC AGT CGT AG respectively for *HOXA5*, and 5′-GGA GTA AAA TAG GTG AAA GT-3′, 5′-GCC CTT CCC CAA CCT C-3′, 5′-GGT TTT TTT TTT TTA TTA CGT ATT-3′ and GTC AGT TGG TGA respectively for *WNT5A.* The sequence of the universal biotinylated PCR primer was 5′-GGG ACA CCG CTG ATC GTT TA-3′.

### Statistical analysis

Data from a total of 84 CpG sites contained within 39 X-chromosome genes on the array were removed from the analysis to eliminate gender-specific bias. Thus, 1,421 probes across 768 genes were included for the analyses. All statistical analyses were done in R version 2.14.2 at 5% significance level unless otherwise stated (The R Foundation for Statistical Computing).

The *rpmm* function in the *RPMM* library was used for the identification of methylation subgroups. Recursively partitioned mixture model (RPMM) is a model-based unsupervised clustering approach developed for beta-distributed DNA methylation measurements that lie between 0 and 1 [34]. A fanny algorithm was used for initialization and level-weighted version of Bayesian information criterion (BIC) as a split criterion for an existing cluster as implemented in the R-based RPMM package [35]. For the purpose of comparison, classification of tumor samples was also performed with the optimal number of clusters determined using the Calinski-Harabasz pseudo F-statistic [36], and the robustness evaluated by bootstrap resampling analysis (n = 1000). Graphical representations of the β-values were achieved by the *heatmap.plus* function with the *gplots* and *heatmap.plus* libraries.

Identification of CpG sites that were differentially methylated between tumors and matched tumor-adjacent gastric tissues was performed using the paired sample t-test, while that between methylation subgroups was done using the ANOVA-test. A Benjamini and Hochberg false discovery rate (FDR) cut-off of 0.001 was used, with a Supplementary filter of a minimum difference of 0.15 in the average β-value between the two groups, as described earlier [37,38]. The associations of methylation subgroups with clinicopathological and molecular factors were compared with the likelihood ratio or Fisher’s Exact test where appropriate. Average methylation level and frequency of methylation (on binarized data) across methylation subgroups was compared using ANOVA and the likelihood ratio test respectively.

All statistical analyses were carried out using the β-value as a continuous variable unless specified otherwise. When β-values were binarized, a methylated threshold of 0.297 was used [39]. Pathway analysis was performed for KEGG pathway mapping using DAVID with a threshold of EASE score ≤ 0.05 [40-42].

## Results

### DNA methylation patterns in gastric tumor and *tumor-adjacent* tissues

Unsupervised hierarchical clustering of methylation levels from all 1,421 autosomal CpG sites in 60 tumor samples revealed five distinct subgroups [Additional file 1]. No distinct subgroups were observed for the corresponding tumor-adjacent gastric tissues, with RPMM analysis computing the number of distinct subgroups was equal to the number of cases. These findings support the occurrence of non-random methylation events in tumorigenesis.

A total of 219 CpG sites (185 hypermethylated and 34 hypomethylated) in 147 unique genes were significantly differentially methylated between tumor and tumor-adjacent gastric tissue (FDR = 0.001), with the top three CpG sites located in *HOXA5*, *SFRP1* and *CCNA1* [Additional file 2]. Analysis by DAVID revealed that “Pathways in cancer”, the “Hedgehog signalling pathway” and “Cytokine-cytokine receptor interaction” were the top three significant pathways revealed by genes with tumor-specific methylation in GC (Table 1).

**Table 1:** KEGG pathways of significantly differentially methylated genes

**Pathway**	**Entry**	***P *** **value (EASE score)**	**Genes**
**Different between tumor and tumor-adjacent gastric tissue**			
Pathways in cancer	hsa05200	0.0000014	DCC, WNT5A, FGF5, RET, FGF8, FLT3, PPARG, TGFB3, FGF12, MMP2, GLI3, TGFB2, WNT2, SMO, CSF3R, HHIP, CCNA1, FGF2, FGF3
Hedgehog signaling pathway	hsa04340	0.0015474	WNT2, WNT5A, SMO, HHIP, GLI3, BMP6
Cytokine-cytokine receptor interaction	hsa04060	0.0019703	LIF, FLT1, FLT3, FLT4, IFNG, TGFB3, CSF3R, NGFR, KDR, TNFSF8, EPO, TGFB2
Basal cell carcinoma	hsa05217	0.0096687	WNT2, WNT5A, SMO, HHIP, GLI3
Hematopoietic cell lineage	hsa04640	0.0098799	CD34, FLT3, CD2, CSF3R, MME, EPO
MAPK signaling pathway	hsa04010	0.0201135	FGF5, FGF8, RASGRF1, NTRK2, MOS, TGFB3, FGF12, FGF2, FGF3, TGFB2
Melanoma	hsa05218	0.0229304	FGF5, FGF8, FGF12, FGF2, FGF3
TGF-beta signaling pathway	hsa04350	0.0437881	IFNG, TGFB3, THBS2, BMP6, TGFB2
Regulation of actin cytoskeleton	hsa04810	0.0456631	FGF5, FGF8, TIAM1, INS, MOS, FGF12, FGF2, FGF3
Axon guidance	hsa04360	0.0471078	DCC, EPHA7, EPHA8, SEMA3C, FES, SLIT2
**Different between tumor subtype H and L**			
Pathways in cancer	hsa05200	0.0000012	DCC, WNT5A, FGF5, FGF8, RET, FLT3, MMP2, GLI3, TGFB2, WNT2, SMO, HHIP, CCNA1, FGF3
Hedgehog signaling pathway	hsa04340	0.0000843	WNT2, WNT5A, SMO, HHIP, GLI3, BMP6
Basal cell carcinoma	hsa05217	0.0010110	WNT2, WNT5A, SMO, HHIP, GLI3
Cytokine-cytokine receptor interaction	hsa04060	0.0194278	FLT1, FLT3, FLT4, NGFR, KDR, EPO, TGFB2

### GC subgroups revealed by tumor-specific CpG methylation

**Figure 1:**
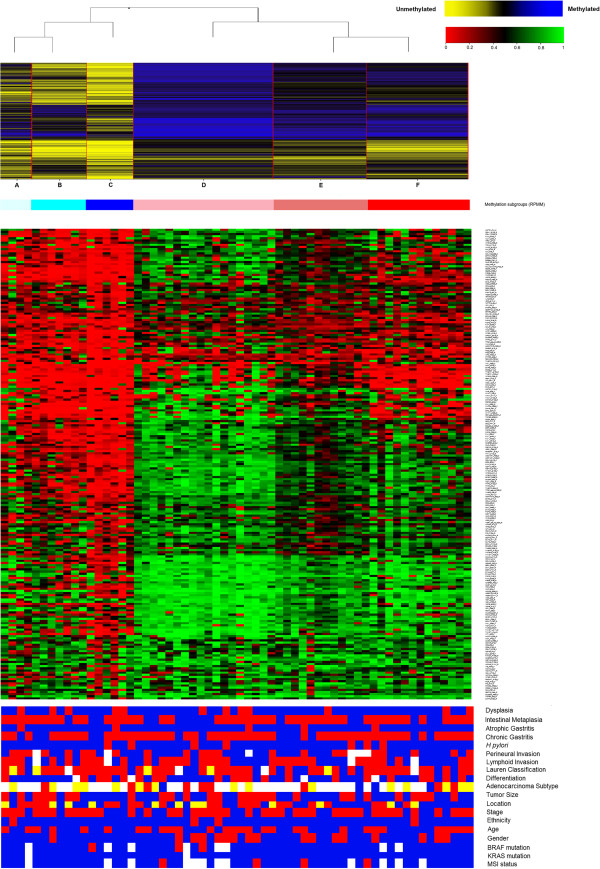
**Cluster diagram of 219 tumor-specific CpG sites (rows) in 60 GC tumor samples (columns).** The RPMM tree and clusters (labelled A-F) under the two major subtypes (A-C and D-F) are shown at the top of the Figure. Clinicopathological and molecular features are shown below the cluster diagram. White rectangles are cases with missing data. History of dysplasia: yes (red), no (blue); History of intestinal metaplasia: yes (red), no (blue); History of atrophic gastritis: yes (red), no (blue); History of chronic gastritis: yes (red), no (blue); *H. pylori* status: yes (red), no (blue); Perineural invasion: yes (red), no (blue); Lymphoid invasion: yes (red), no (blue); Lauren classification: diffuse (blue), intestinal (red), mixed (yellow); Differentiation: poor (blue), moderate (red); Adenocarcinoma subtype: mucinous (blue), signet ring (red), tubular (yellow); Tumor size: >4.5 cm (median) (red), ≤4.5 cm (blue); Location: distal 1/3 (blue), middle 1/3 (red), proximal 1/3 (yellow); Stage: III/IV (red), I/II (blue); Ethnicity: Non-Chinese (red), Chinese (blue); Age: >71 years (median) (red), ≤71 years (blue); Gender: female (red), male (blue); *BRAF* mutation: mutant (red), wildtype (blue); *KRAS* mutation: mutant (red), wildtype (blue); MSI status: MSI (red), MSS (blue).

Unsupervised clustering of the 219 tumor-specific CpG methylation sites by RPMM analysis revealed six GC clusters, denoted A-F (Figure 1). Bootstrap resampling analysis (n = 1,000) indicated two groups were the mode (91%) for the optimum number, hence the six clusters were consolidated into two major subtypes denoted as L (low methylation; clusters A-C; 28% of cases) and H (high methylation; clusters D-F; 72% of cases). In support of this classification, the mean methylation value (β-value) in subtype H tumors was twice that observed in subtype L (0.603 *vs*. 0.305, respectively; *P* < 0.001). Using a β-value threshold of ≥0.297 to binarize DNA methylation levels as described previously [39], H tumors also showed twice as many methylated CpG sites (180/219, 82%) compared to L tumors (89/219, 40%; *P* < 0.001). When RPMM was performed on the matched tumor-adjacent gastric mucosa using the tumor-specific CpG sites, the samples did not cluster according to the subtypes (L or H) of their corresponding paired tumors [Additional file 3]. This suggests the methylation patterns observed in tumors did not pre-exist in tumor-adjacent gastric tissue and were likely to result from somatic events.

Of the 219 CpG sites showing differential methylation between tumor-adjacent gastric and tumor tissue, 114 were also significantly different between the H and L tumor subgroups (FDR = 0.001) and all of these were hypermethylated in H but not L tumors [Additional file 4]. The three CpG sites showing the most significant difference in methylation level between H and L tumors were located in *SEZ6L*, *FLT4* and *ALK*. Interestingly, the four most significant KEGG pathways identified by the differentially methylated genes between tumor and tumor-adjacent gastric tissues were also identified by the differentially methylated genes between H and L tumors (Table 1).

Associations between clinicopathological and molecular features and the H and L GC subtypes defined by methylation are shown in Figure 1 and Additional file 5. GC from female patients were almost all subtype H (16/17, 94%) and this was significantly higher than for male patients (27/43, 63%; *P* = 0.024). No significant associations were observed between the H and L subgroups and any of the other clinicopathological or molecular features of GC.

### Methylation status and genome location, polycomb occupancy and histone modification

CpG sites that were differentially methylated between tumor and tumor-adjacent gastric tissue as well as between tumor subtypes H and L were referred to as Group HG (114 loci in 78 genes). CpG sites that were differentially methylated between tumor and tumor-adjacent gastric tissue, but not between tumor subtypes H and L, were referred to as Group LG (105 loci in 69 genes), while CpG sites not differentially methylated between tumor and tumor-adjacent gastric tissue were classified as Group NG (1,202 loci in 626 genes).

**Figure 2:**
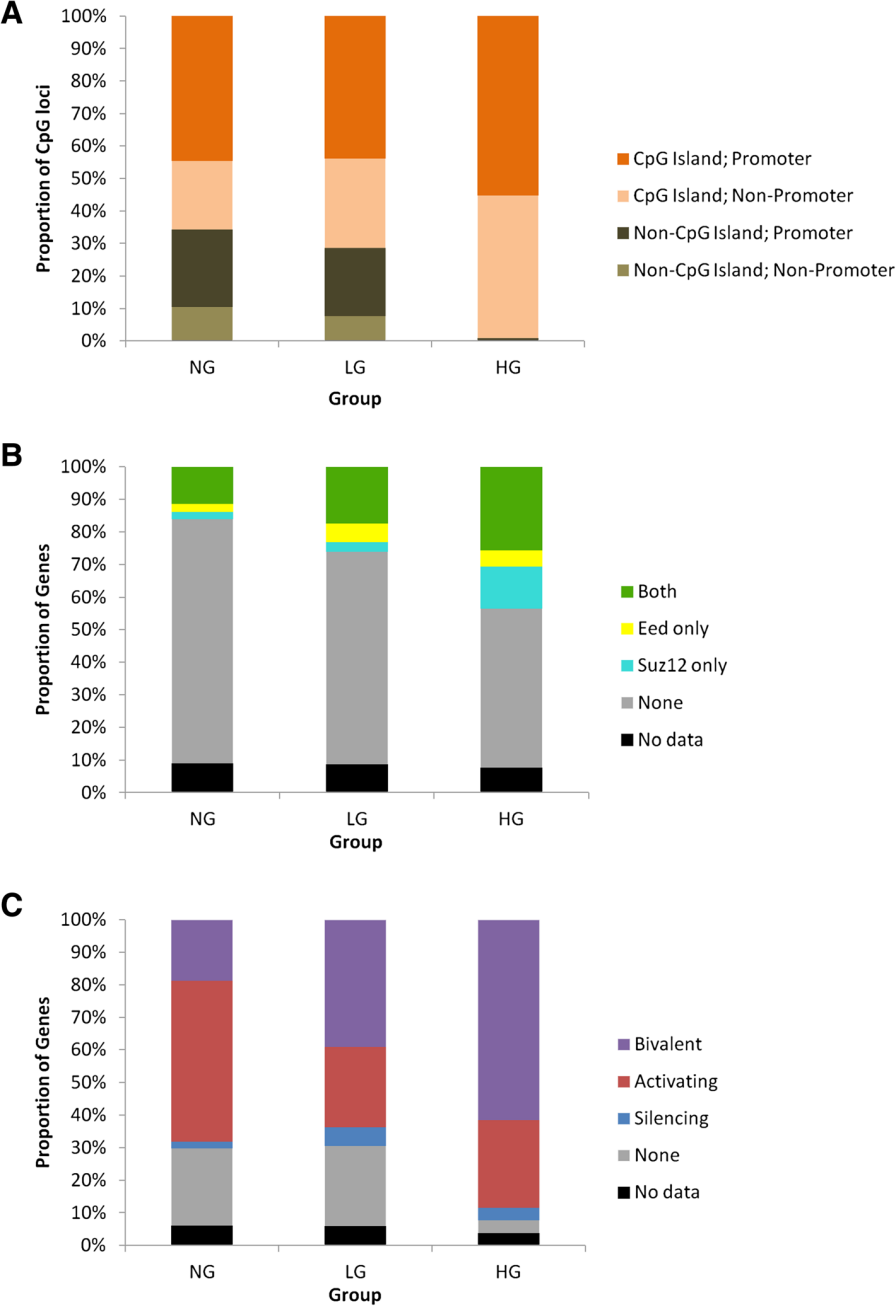
**Distribution of (A) genomic location in CpG island and gene promoter regions (B) PRC2 occupancy, and (C) Histone modifications across tumor subgroup-specific gene groups.** Bivalent: H3K4 + H3K27+; Activating: H3K4 + H3K27-; Silencing: H3K4-H3K27 +.

The distribution of CpG loci within CpG islands and promoters according to their annotation in the GoldenGate information sheet is displayed in Figure 2A for the NG, LG and HG groups. The proportion of CpG loci located within CpG islands increased progressively from NG (66%) to LG (71%) and HG (99%), with the differences between HG and LG (*P* < 0.001) and HG and NG (*P* < 0.001) being significant. This result shows that almost all differentially methylated CpG sites in the H group of GC were located within CpG islands.

Polycomb receptor complex-2 (PRC2) occupancy of gene promoters by the components Suz12 and Eed has been linked to susceptibility to methylation in human embryonic stem cells [43]. In agreement with this, the proportion of genes with occupancy of both factors steadily increased from Groups NG (10%) to LG (19%) and HG (28%) (Figure 2B). The differences between groups NG and LG (*P* = 0.034) and between NG and HG (*P* <0.001) were significant.

The H3K4 and K3K27 trimethylation status in human embryonic stem cells has been shown to reflect modes of gene regulation in differentiated cells, namely constitutive expression (H3K4+ K3K27-), constitutive repression (H3K4-K3K27+), and bivalent, or “primed” for repression and expression (H3K4+ K3K27+) [44]. A significantly higher proportion of Group HG genes showed H3K4+ K3K27+ bivalent marks (62%) compared to Group LG (39%, *P* = 0.008) and Group NG genes (19%, *P* < 0.001) (Figure 2C).

### CpG sites methylated according to *H. pylori* status

None of the CpG sites investigated showed significantly different methylation between tumor samples of HP+ and HP- patients. However, comparison of the tumor-adjacent gastric tissue between HP+ and HP- patients revealed 8 differentially methylated CpG sites located within 7 genes (CCNA1, CSPG2, DAB2IP, DIO3, FLT1, STAT5A and TWIST1) [Additional file 6]. All 8 sites were hypomethylated in HP+ compared to HP- cases.

### Verification of differential methylation

To verify the robustness of the observed differential methylation, pyroseqeuencing was performed to quantify methylation at the same CpG site of the top differentially methylated gene (*HOXA5*) and a gene from the top differentially methylated pathway (*WNT5A*, hedgehog signalling) in an independent series of tumor and matched tumor-adjacent gastric tissues from 60 subjects with GC. The higher level of methylation in tumors compared to tumor-adjacent gastric tissues that was observed in GoldenGate analysis was observed again for both *HOXA5* (mean difference = 16.4%, P < 0.001 by paired t-test) and *WNT5A* (20.0%, P < 0.001) in this independent series (Additional file 7).

## Discussion

Candidate gene studies have so far identified 77 genes that are differentially methylated between normal and malignant gastric tissue [27]. In the present work, the methylation of 1,421 autosomal CpG sites located within 768 cancer-related genes was evaluated in 60 pairs of GC and matched tumor-adjacent gastric tissue. A total of 219 CpG sites within 147 genes were found to be differentially methylated. Only 27/77 (35%) of the genes previously identified as being differentially methylated in the candidate gene studies were included in the GoldenGate methylation arrays used here. Hence, with the exception of 6 genes (CHFR, DAB2IP, DLC1, SFRP1, TCF4 and TFPI2), almost all of the 147 genes identified in the present study are novel methylation markers for GC that could be investigated further for potential roles in gastric tumorigenesis and for early screening.

GoldenGate methylation arrays have previously been used for comprehensive methylation studies of several cancer types including colorectal, head and neck, renal, breast and non-small cell lung cancer [10,39,45-47]. They have also been used to study non-cancerous gastric mucosa with respect to *H. pylori* infection and the presence of malignant tissue [48]. However, the current study is the first to apply GoldenGate methylation arrays to investigate differential methylation between GC and matched tumor-adjacent gastric tissues. We believe that the high-quality tissues used in our study allows us to add value to the available scientific knowledge. Specifically, this unbiased, genome-wide approach revealed the existence of six methylation subgroups contained within two distinct clusters that comprised 28% (L) and 72% (H) of GC tumors (Figure 1). The mean methylation level of CpG loci in H tumors was twice that of L tumors. Compared to L tumors, H tumors were significantly over-represented with female patients (37% *vs*. 6%). Despite the small number of MSI cases (n = 4), a trend was also observed for more frequent association of the MSI phenotype with H tumors (9% *vs*. 0%).

The associations of female gender and MSI with the high methylation (H) subgroup of GC are in line with CIMP+ CRC, where these associations are reported consistently. The observation that hypermethylated CpG loci in the H subgroup are almost exclusively located in CpG islands (Figure 2A) is also consistent with the definition of CIMP and with previous findings in CIMP+ CRC [13-16,49]. In general, however, the evidence in support of a distinct CIMP+ GC subgroup has so far been unconvincing. Highly methylated GC subgroups have shown contradictory associations with the stage of disease [18,19,25,26,50], histological type [17-19,22,50] and patient survival [17,19,20,50]. Reported associations with older age [51], proximal tumor location [18] and poor differentiation [19] have not been confirmed by others, although more consistent associations have been reported with Epstein Barr virus infection [17,18,50], lymph node metastasis [25,51] and MSI [20,52].

A likely explanation for the inconsistent results to date on CIMP in GC is that studies have been limited to a small number of genes used different methylation assays and thresholds [5]. Array-based studies have enabled a more comprehensive analysis of the DNA methylome. In addition to the present study using GoldenGate methylation arrays, Kim et al. recently published results using Infinium HumanMethylation450 BeadChip arrays that evaluate almost 500,000 CpG sites [53]. They reported a CIMP+ subgroup in 11 of 30 (37%) GC samples analysed. These tumors displayed a higher frequency of oncogene mutations including *KRAS* and *PIK3CA*. Zouridis et al. also recently published their results on 203 GC obtained using Infinium HumanMethylation27 BeadChip arrays that evaluate 27,578 CpG sites [54]. These workers reported a CIMP+ subgroup that comprised a similar proportion (35%) to that reported by Kim et al. but was considerably smaller than the H subgroup found in the current study (72%). The CIMP+ subgroup described by Zouridis et al. was also characterized by younger patient age and worse survival. A possible source of bias in our study was that all CpG sites evaluated by the Illumina GoldenGate Methylation Cancer Panel I array were within cancer-related genes. Moreover, only two or less CpG sites were evaluated for most (86%) of these genes. Together with the larger Illumina Infinium BeadChip arrays, next generation sequencing will enable even more comprehensive profiling of the methylome in GC. However, this does not necessarily imply that a distinctive CIMP+ GC subgroup will be identified in a consistent fashion.

*HOXA5* was found here to be the most differentially methylated gene between gastric tumor and tumor-adjacent gastric tissue (Additional file 2), with the observation of a higher methylation level in tumor replicated in an independent series using a different experimental technique (*P* < 0.001; pyrosequencing). The *HOX* gene family, of which *HOXA5* is a member, is known to play important roles in embryonic development and adult cell differentiation [7]. *HOXA5* is temporally expressed in the mesenchymal component of the developing gut [55] and a loss of *HOXA5* function can perturb intestinal maturation in mice [56]. Hypermethylation of *HOXA5* has been reported in several cancer types [57-61] and is associated with decreased expression [59,62]. The present study is the first to our knowledge to report *HOXA5* methylation in GC. This finding warrants further functional studies to determine whether methylation-induced silencing of *HOXA5* is a driver event for gastric tumorigenesis. The second most differentially methylated gene observed here, *SFRP1*, has previously been reported to be methylated in over 90% of primary GC [63].

Recursive partitioning identified a single CpG site within *SEZ6L* whose methylation status could differentiate the L and H GC subgroups. *SEZ6L* methylation has previously been reported in GC [23] and CRC [64]. *SEZ6L* methylation in the gastric mucosa of non-GC subjects has also been associated with *H. pylori* infection [48]. The role of this gene in gastric tumorigenesis is currently unclear, although it has been implicated as a risk factor for lung cancer [65,66].

The associations between methylated genes, polycomb occupancy and H3K4/H3K27 modifications observed here for GC (Figure 2B and C) and elsewhere for other cancer types suggests that aberrations in chromatin regulation could underlie the hypermethylation phenotypes observed in cancer. The recent introduction of standardized methylation assay platforms with genome-wide coverage, such as the Illumina Infinium BeadChip arrays, should allow this area to be investigated in much more detail in future studies.

Aberrant methylation of gastric mucosa has been implicated in the elevated risk of GC in HP-infected individuals [67,68]. Other studies have reported differentially methylated genes between HP- and HP+ GC tissue [48,69]. The current study did not find any genes that were differentially methylated between HP- and HP+ GC tissue, but did find 8 genes that were hypomethylated in the tumor-adjacent gastric tissue of HP+ GC patients [Additional file 5]. Interestingly, amongst these 8 genes were *DAB2IP* and *TWIST1*, both of which have been implicated in gastric tumorigenesis [70,71]. *STAT5A* was also previously observed to be hypomethylated in HP+ compared to HP- tissue from non-GC subjects [48], thus mirroring the present results in GC patients. *CDH1*, *FLNC* and *HAND1* were previously reported to be methylated in HP+ GC tissues [67-69] using the GoldenGate array, but were not differentially methylated in the current study. This may due to the use of continuous versus binary values for methylation and to the thresholds used for statistical testing. Differential gene methylation in normal gastric mucosa between HP+ and HP- individuals may reflect the fact this pathogen is an initiating factor in the neoplastic transformation of gastric mucosa.

## Conclusions

Methylation analysis of 1,421 CpG loci led to the identification of 147 genes that were differentially methylated in GC. Two major subgroups of GC were identified according to unbiased analysis of methylation levels. Tumors with high levels of methylation (subgroup H) shared some features consistent with CIMP in CRC. The methylation status of a single CpG site in *SEZ6L* was sufficient to allow absolute discrimination of the L and H subgroups of GC. Methylated genes in subtype H were characterized by higher frequencies of polycomb occupancy and H3K4+/H3K27+ bivalent marks, thus providing evidence of links between the hypermethylated phenotype and chromatin dysregulation. Further investigations of large population-based series are required to validate these findings and to assess the clinical utility of subgroups defined by methylation status.

## Abbreviations

BIC: Bayesian information criterion; CIMP: CpG island methylator phenotype; CRC: Colorectal cancer; DSS: Disease-specific survival; DFS: Disease-free survival; FDR: False discovery rate; FFPE: Formalin-fixed and paraffin-embedded; GC: Gastric cancer; H. pylori: *Helicobacter pylori*; MSI: Microsatellite instability; MSS: Microsatellite stable; OS: Overall survival; RPMM: Recursively partitioned mixture model.

## Competing interests

The authors declare that they have no competing interests.

## Authors’ contributions

ML performed all statistical analysis and drafted the manuscript. NL, AV, PLL, NSS, EE, and ZUM carried out the experimental work. CLC, BY, BP, MST, and WPY contributed critical clinical resources, assessment and perspective. BI and RS co-ordinated the study and compiled the manuscript. All authors read and approved the final version of the manuscript.

## Pre-publication history

The pre-publication history for this paper can be accessed here:

http://www.biomedcentral.com/1471-230X/14/55/prepub

## Supplementary Material

Additional file 1 Cluster diagram of 1,421 CpG sites (rows) in 60 gastric tumors (columns). Click here for file

Additional file 2 **List of 219 CpG sites differentially methylated between tumor and matched tumor-adjacent gastric tissues.** Click here for file

Additional file 3 **Cluster diagram of 219 tumor-specific CpG sites (rows) in 60 matched tumor-adjacent gastric tissue samples (columns).** Click here for file

Additional file 4 List of 114 CpG sites differentially methylated between subtypes H and L. Click here for file

Additional file 5 Distribution of clinicopathological and molecular features between subtypes H and L. Click here for file

Additional file 6 **List of eight CpG sites significantly differentially methylated in tumor-adjacent gastric tissue from HP+ and HP- cases.** Click here for file

Additional file 7 **Methylation levels of *HOXA5 *(left chart) and *WNT5A *(right chart) in matched pairs of tumor-adjacent and tumour tissue.** The lines connect methylation levels in matched samples. Click here for file
