# Supplementary figures and images for: DNA methylation subgroups and the CpG island methylator phenotype in gastric cancer: a comprehensive profiling approach
Source: BMC Gastroenterol. 2014 Mar 28;14:55. doi: 10.1186/1471-230X-14-55 (PMC3986689; doi:10.1186/1471-230X-14-55)

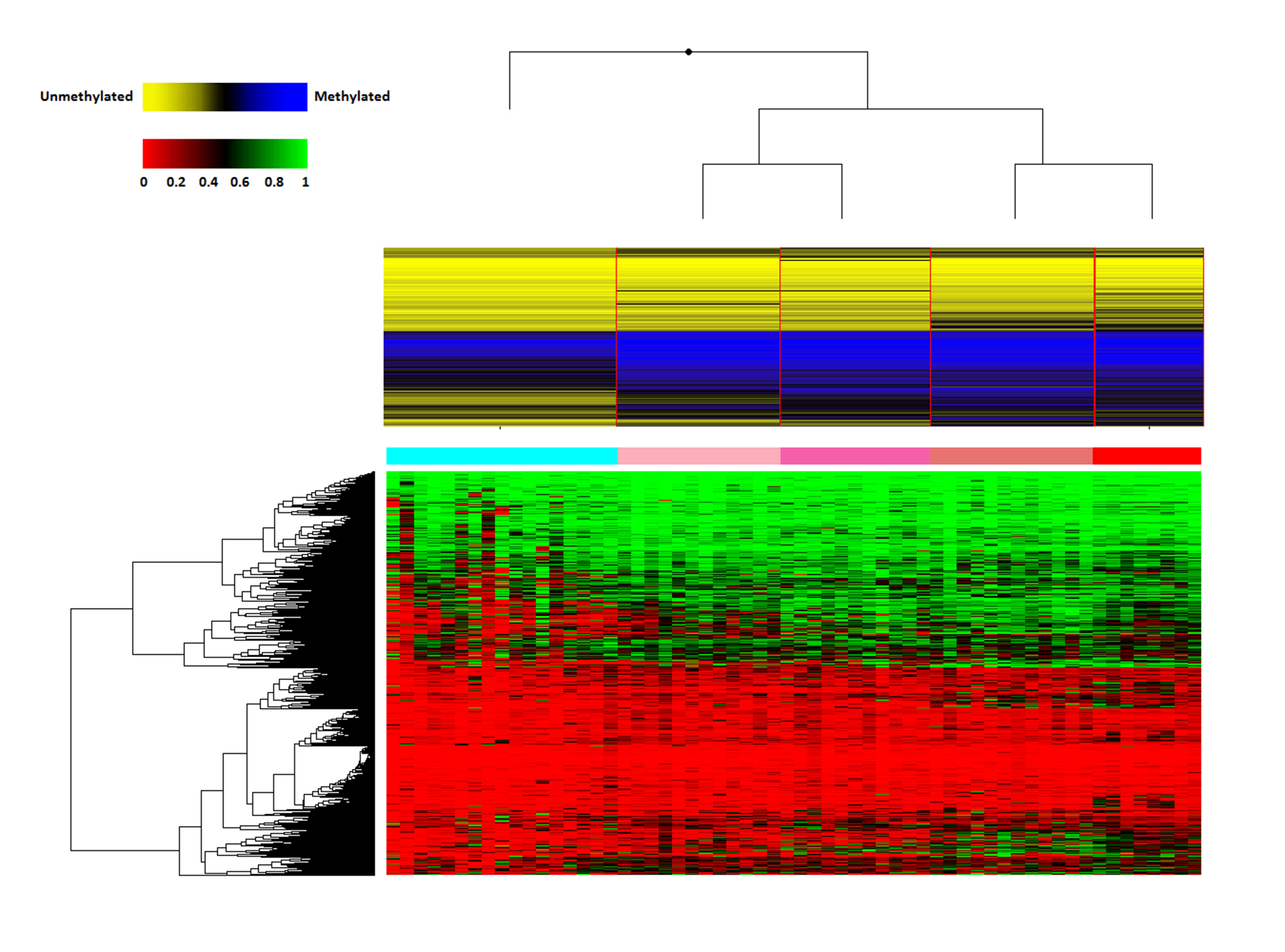

Supplement: Additional file 1 — Cluster diagram of 1,421 CpG sites (rows) in 60 gastric tumors (columns). [file 1471-230X-14-55-S1.tiff]

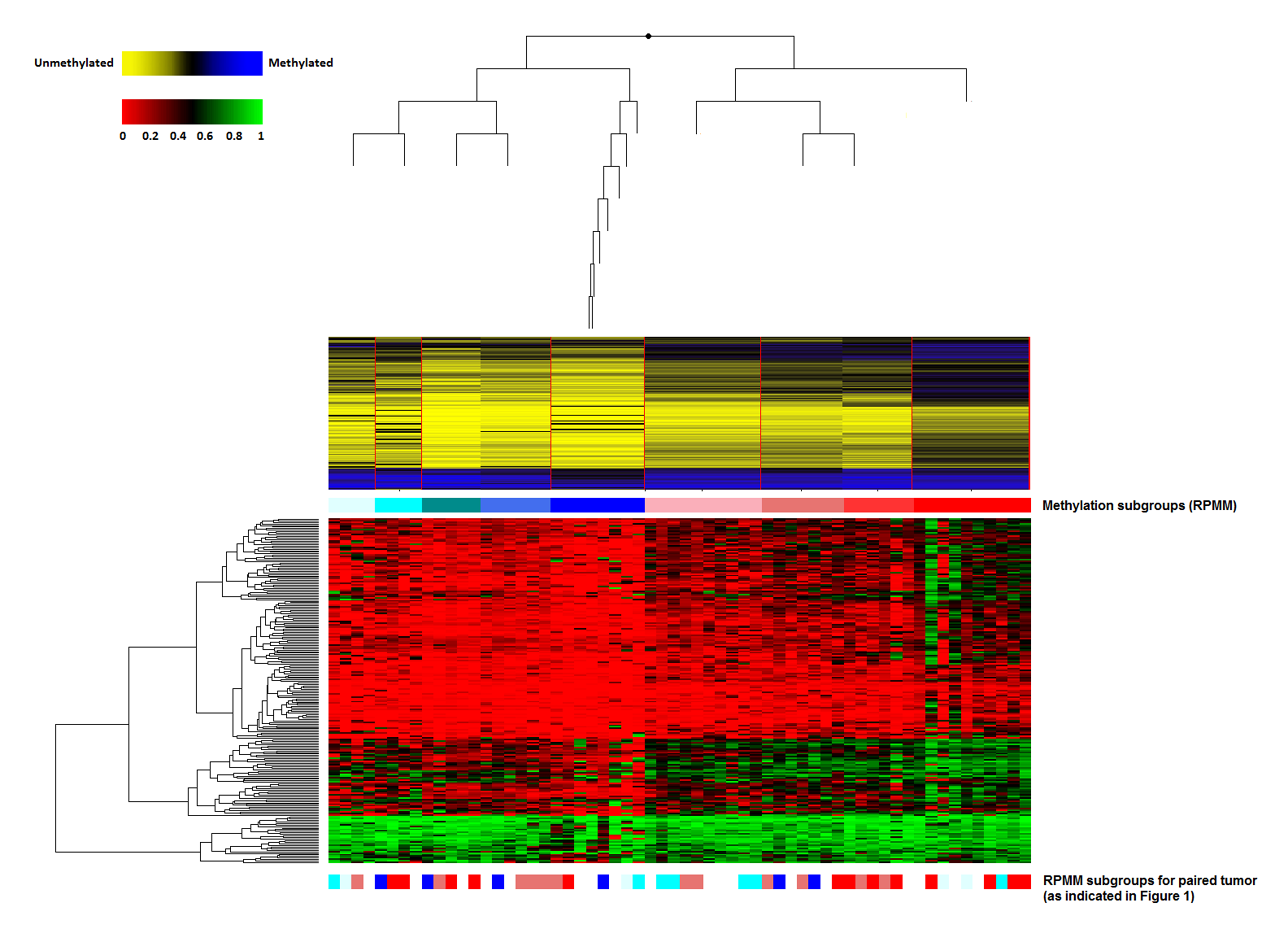

Supplement: Additional file 3 — Cluster diagram of 219 tumor-specific CpG sites (rows) in 60 matched tumor-adjacent gastric tissue samples (columns). [file 1471-230X-14-55-S3.tiff]

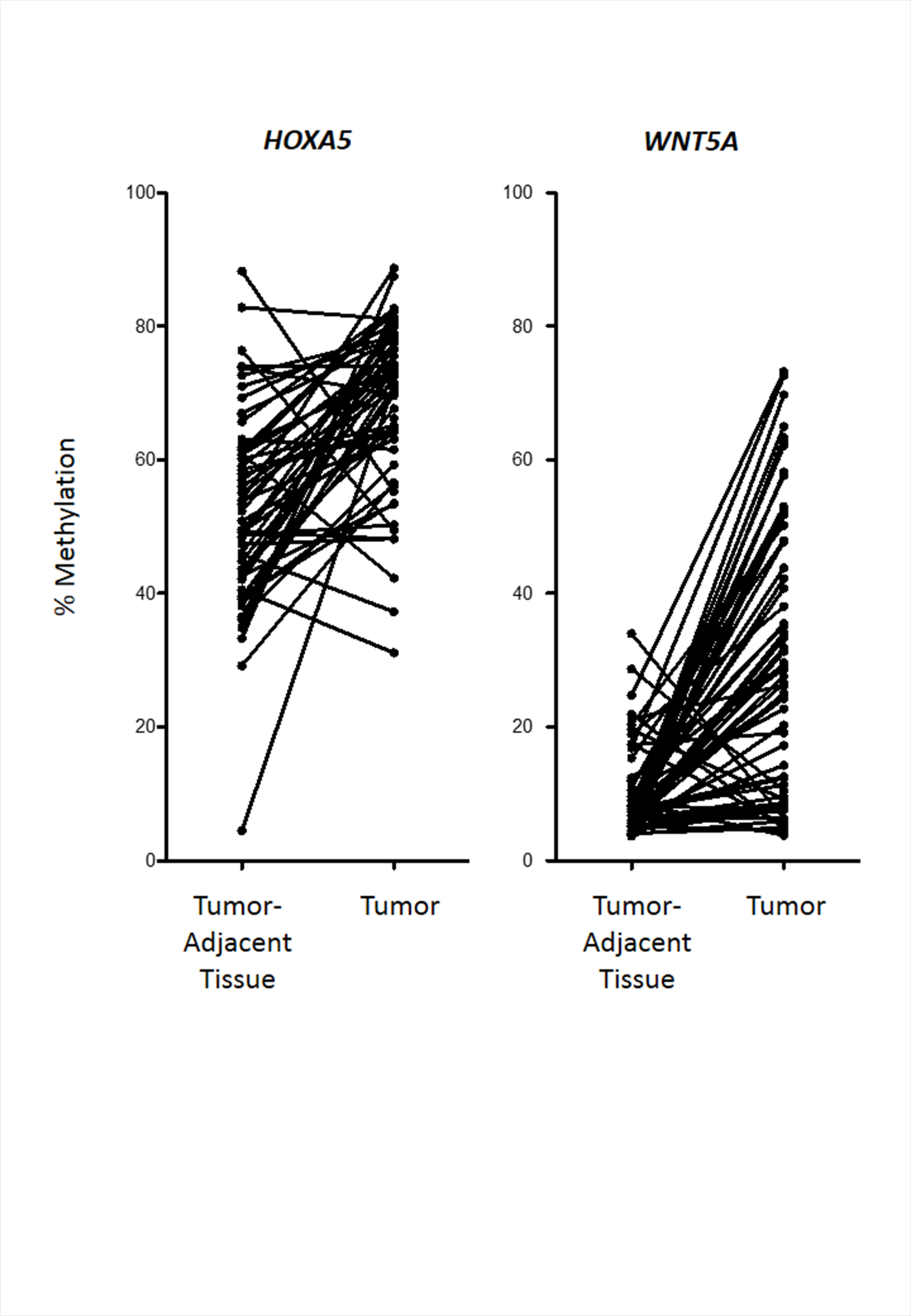

Supplement: Additional file 7 — Methylation levels of HOXA5 (left chart) and WNT5A (right chart) in matched pairs of tumor-adjacent and tumour tissue. The lines connect methylation levels in matched samples. [file 1471-230X-14-55-S7.tiff]
